# Simple SPION Incubation as an Efficient Intracellular Labeling Method for Tracking Neural Progenitor Cells Using MRI

**DOI:** 10.1371/journal.pone.0056125

**Published:** 2013-02-28

**Authors:** Chiao-Chi V. Chen, Min-Chi Ku, Jayaseema D. M., Jiann-Shiun Lai, Dueng-Yuan Hueng, Chen Chang

**Affiliations:** 1 Institute of Biomedical Sciences, Academic Sinica, Taipei, Taiwan; 2 Functional and Micro-magnetic Resonance Imaging Center, Academic Sinica, Taipei, Taiwan; 3 Development Center for Biotechnology, New Taipei, Taiwan; 4 Department of Neurological Surgery, Tri-Service General Hospital, Taipei, Taiwan; 5 Department of Biochemistry, National Defense Medical Center, Taipei, Taiwan; Indian Institute of Toxicology Reserach, India

## Abstract

Cellular magnetic resonance imaging (MRI) has been well-established for tracking neural progenitor cells (NPC). Superparamagnetic iron oxide nanoparticles (SPIONs) approved for clinical application are the most common agents used for labeling. Conventionally, transfection agents (TAs) were added with SPIONs to facilitate cell labeling because SPIONs in the native unmodified form were deemed inefficient for intracellular labeling. However, compelling evidence also shows that simple SPION incubation is not invariably ineffective. The labeling efficiency can be improved by prolonged incubation and elevated iron doses. The goal of the present study was to establish simple SPION incubation as an efficient intracellular labeling method. To this end, NPCs derived from the neonatal subventricular zone were incubated with SPIONs (Feridex®) and then evaluated *in vitro* with regard to the labeling efficiency and biological functions. The results showed that, following 48 hours of incubation at 75 µg/ml, nearly all NPCs exhibited visible SPION intake. Evidence from light microscopy, electron microscopy, chemical analysis, and magnetic resonance imaging confirmed the effectiveness of the labeling. Additionally, biological assays showed that the labeled NPCs exhibited unaffected viability, oxidative stress, apoptosis and differentiation. In the demonstrated *in vivo* cellular MRI experiment, the hypointensities representing the SPION labeled NPCs remained observable throughout the entire tracking period. The findings indicate that simple SPION incubation without the addition of TAs is an efficient intracellular magnetic labeling method. This simple approach may be considered as an alternative approach to the mainstream labeling method that involves the use of TAs.

## Introduction

Cellular magnetic resonance imaging (MRI) has been well-established as a useful approach for tracking neural progenitor cells (NPC) transplanted for therapeutic purposes [1]–[3]. Magnetic cell labeling, which involves incorporating magnetic substances into the intracellular space of cells, is an essential step for this technique [4]–[6]. Among the various MR contrast media, clinically approved dextran coated superparamagnetic iron oxide nanoparticles (SPIONs) are the most extensively used agents for tracking cells owing to their safety, clinical applicability, and effectiveness [1], [5], [7]–[9].

It has long been viewed that approved SPIONs in their native unmodified form is not efficient for intracellular labeling [9]–[12]. Consequently, transfection agents (TAs) are conventionally used in combination with SPIONs to facilitate the labeling [2], [5], [9], [13]. However, the utilization of TAs presents some major obstacles to the clinical applicability of cellular MRI. Most TAs are cationic lipids or proteins, and form complexes with SPIONs via electrostatic interactions. These complexes, once degraded [14], become potentially toxic to the transplanted tissue or organism via oxidative stress and induced apoptosis [14]–[17]. In addition, the TA complexes tend to form aggregates in the culture medium. During labeling, the aggregates are very likely to adhere to cell membranes without being internalized into cells [18], [19]. Once the cells are transplanted, the non-internalized aggregates may be detached from the NPCs and lead to an inaccurate representation of cell distribution.

In spite of abundant literature with regard to SPION-labeling using TAs [3], [5], [6], [9], [10], simple labeling of NPCs free of TAs has not been studied thoroughly. It was generally believed that, although intracellular internalization of SPIONs occurs spontaneously [11], it is not efficient enough to load a significant amount of particles [6], [10], [20]. For instance, 100% labeling was achieved when the cells were incubated with the SPION-TA complexes at 25 mg Fe/mL for two hours whereas SPION alone produced undetectable labeling under the same condition [9]. However, evidence also indicates that simple SPION incubation is not invariably ineffective [19]. It truly depends upon the incubation time and the iron concentration. A concentration as high as 4.17 mg Fe/mL cells for 4 hours rendered the labeled cells detectable on MRI [11]. Alternatively extending the incubation time up to 24 hours improved the labeling to an observable level given a concentration of 50 µg Fe/mL [20]. Prolonged incubation and elevated iron doses helped increase the intracellular loading of SPIONs. But overexposure to high iron levels for extended time may decrease cell survival and proliferation [3]. Optimization of these two factors is prerequisite to the determination of the labeling efficiency of the simple SPION incubation method.

The present study aims to demonstrate simple SPION incubation as an efficient intracellular labeling method for NPCs. Since it only uses the approved SPIONs, it is readily clinical applicable, and thus can be considered as an alternative that avoids the complications of TAs. To assess the efficiency and effects of the proposed labeling method, NPCs derived from the neonatal subventricular zone (SVZ) were incubated with SPIONs (Feridex®) and then evaluated *in vitro* with regard to the labeling efficiency, intracellular internalization, oxidative stress, apoptosis, viability, differentiation, and MR detectability. The findings arising from the present study support an alternative approach to the mainstream labeling method that involves the use of TAs.

## Materials and Methods

### Ethics Statement

The Institutional Animal Care and Use Committee (IACUC) of the Institute of Biomedical Sciences, Academia Sinica approved the procedures performed in the present study. In compliance with the regulations, extreme caution was taken to ameliorate and minimize suffering of the animals.

### Primary NPC Culture

Brains were removed from neonatal Sprague-Dawley rats (postnatal day 0) by decapitation. The brain tissue around the SVZ was excised by fresh scalpel blades and propagated as free-floating aggregates in serum-free DMEM-F12 containing 20 ng/mL epidermal growth factor (EGF) 6.28 ng/mL progesterone, 10% hormone mixture, 1 M HEPES, 30% d-glucose, 100 U/mL penicillin, and 100 mg/mL streptomycin. The aggregates were dissociated by mechanical trituration with a fire–polished Pasteur pipette and then passed through a 40 µm cell strainer. The cells were plated on uncoated 75 T flasks at a density of 7.5×10^3^ cells/ml, and were allowed to develop into neurospheres in a humidified atmosphere with 5% CO_2_ at 37°C. Seven days after plating, the primary neurospheres were dissociated and then reseeded into a fresh medium. The NPC culture protocol was adopted according to the methods described previously [21], [22].

**Figure 1:**
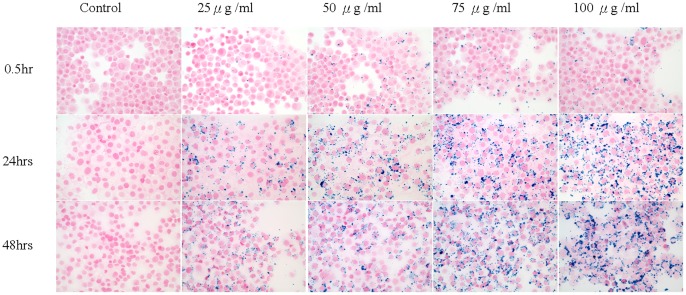
The labeling efficiency is determined by the incubation time and iron concentrations. Prolonged incubation and high iron concentrations resulted in more labeling. In the present study, the optimized incubation condition for NPCs was 75 µg/ml over 48 hours. Higher concentrations than 75 µg/ml tended to cause undesired aggregation (see the 100 µg/ml panel).

The phenotype of the SVZ cells was identified by immunostaining. As shown in Fig. 1A, a majority of the cells within the spheres were positive for nestin, a commonly used marker for neural progenitors [23]. The immunohistochemical staining was performed as follows. P1 neurospheres were cytospun onto glass slides. After fixation in 4% paraformaldehyde for 1 hour at room temperature (RT)., the slides were incubated with mouse anti-rat nestin (1∶100; Stemcell technologies Inc., Vancouver, Canada) in PBS containing 0.1% Triton X and 10% normal goat serum overnight at 4°C, followed by incubation in rhodamine conjugated goat anti-mouse IgG (1∶200; Dako, Denmark) at RT for 1 hour. The cells were further stained with DAPI (100 ng/mL; Dako, Denmark). A fluorescent microscope was used to image the staining. Culture media and supplements were purchased from Gibco (Grang Island, USA). Chemicals were from Sigma-Aldrich (St. Louis, USA).

### Magnetic Cell Labeling

SPION (Feridex IV, Berlex, USA) were added directly to the NPC culture at concentrations of 0, 25, 50, 75, and 100 µg Fe/ml. No transfection agent was used. After 0.5, 24, or 48 hrs hours of incubation, cells were washed three times in PBS to remove excessive SPION and used for subsequent assays. The day on which the labeling procedure was completed was designated as day 0.

### Prussian Blue (PB) Staining

Neurospheres or 2×10^5^ NPCs were cytospun onto glass slides, fixed with 10% formalin for 20 mins and then incubated with a 1∶1 mixture of 20% HCl and 2% potassium ferrocyanide for a further 20 mins. Slides were counterstained with nuclear fast red for 15 mins. The percentage of PB stained cells out of total counted cells was calculated. A cell was considered PB stained if the blue precipitate was found within the cytoplasm. The evaluation was performed on ∼200 cells selected randomly, and repeated three times. The quantification was performed using an inverted microscope at the magnification of 100. The counting was done manually.

### Electron Microscopy

Electron microscopy was used to identify the internalization of SPION in the labeled cells. The cells were pelleted and fixed in 2% buffered glutaraldehyde for 1.5 hours before being washed three times in PBS, followed by 1% osmium tetroxide for 1.5 hours in the dark. A total of 20 samples were prepared and each sample contained 10–20 cells. Ultrathin sections were mounted onto slot grids for viewing using a JEM 1200EX transmission electron microscope (TEM).

### Inductively Coupled Plasma Atomic Emission Spectroscopy (ICP-AES)

ICP-AES measured the average iron content in cells. SPION-labeled cell pellets at a density of 1×10^6^ cells/ml were digested by 1 ml of HCl in a 70°C water bath for 30 mins. The mean cellular SPION content was expressed in the unit of pg per cell. Figures show mean ± standard deviation (SD).

### Relaxometry with MRI

The relaxometry experiments were performed on a 4.7T spectrometer (Biospec 47/40, Bruker, Germany) with an active shielding gradient of 200 mT/m in 80 µs, capable of maximum 20 G/cm with a slew rate 2500 T/m/s. A birdcage coil with a 72 mm inner diameter was used for radio frequency excitation and signal reception. Relaxometry was done on single cells dissociated from neurospheres. 200 µl of SPION-labeled or unlabeled control cell suspensions were imaged at a density of 3 × 10^5^/100 µl solidified in 1% agarose in syringes. T2* maps were generated using a fast low angle shot gradient (FLASH) with field of view (FOV) = 4×4 cm; repetition time (TR) = 2000 ms; echo times (TEs) from 2.5 to 52 ms with increments of 5.5 ms; number of excitations (NEX) = 1; flip angel = 30^o^; resolution = 256×256. T2 maps were generated using a multi spin-echo sequence with field of view (FOV) = 4×4 cm; TR = 4000 ms; TEs = 10, 20 to 200 ms with increments of 20 ms; NEX = 1; resolution = 256×256. T2* and T2 maps were generated by fitting the signal intensity data of the various TEs for the exponential decay curves on a voxel by voxel basis using MRvision (MRVision Co., Menlo Park, CA, U.S.A.). The two parameter fits to monoexponential models were used. R2* and R2 were calculated as the inverse of T2* and T2, respectively.

### The MTT Assay

(3-(4,5-Dimethylthiazol-2-yl)-2,5-diphenyltetrazolium bromide (MTT) assay kit (Roche, Mannheim, Germany) was used to determine cell viability. Following SPION labeling, 1000 NPCs were grown in 96-well plates with fresh culture media. The cells were added with MTT reagent (final concentration 0.5 mg/mL) and then incubated for 4 hours, followed by the addition of 100 µL stabilization reagent with overnight incubation. The absorbance of the samples was measured at 570 nm for the purple formazan product and 750 nm for reference. Data reported for each sample represent the mean difference between absorbance at 570 nm and at 750 nm for six duplicate wells.

### The Reactive Oxygen Species (ROS) test

Oxidative stress was assessed by reactive oxygen species (ROS) production in SPION labeled cells at different time points. The oxidant-sensitive probe 5-(and 6-)-chloromethyl- 2′7′-dichlorodihydrofluorescein diacetate acetyl ester (CM-H_2_DCFDA; Molecular Probes, Eugene, USA) was used to determine the intracellular levels of ROS. Both SPION-labeled and unlabeled cells were collected and resuspended in PBS at 1×10^6^ cells/mL. CM-H_2_DCFDA was added at a final concentration of 10 µM and cells were incubated for 1hour at 37°C, followed by being measured by a fluorescence microplate reader system using 495 nm wavelength for excitation and 525 nm for emission (Device, Sunnyvale, USA). Oxidative stress was represented by the difference between the mean intensity of five wells of reagent-loaded cells and autofluorescence.

### The Annexin V Apoptosis Assay

10^6^ cells were resuspended in 100 µL of annexin media (Vybrant apoptosis assay kit no. 2; Molecular Probes, Eugene, USA). 5 µL of fluorescent-labeled annexin V and 2 µL of propidium iodide solution were added to cell suspension, which was then kept at RT for 15 to 20 mins. Flow cytometry was then performed using a fluorescent activated cell sorter (FACS Calibers; Becton Dickinson, USA).

### Neural Differentiation

NPCs differentiate into neurons, astrocytes, and oligodendrocytes when supplemented with the serum or the growth factor. After SPION-labeling, individual sets of NPCs were plated onto ECL cell attachment matrix-coated glass slides in DMEM/F12 supplemented with 1% FBS. At 72 hours after induction, the cells were fixed with 4% paraformaldehyde for 30 mins at RT and then washed three times with PBS. Cells on glass slides were incubated in one of the following primary antibodies, including mouse monoclonal anti-βIII- tubulin (1 µg/mL; AbD Serotec) for neurons, rabbit anti-glial fibrillary acidic protein (GFAP) polyclonal antibody (2.5 µg/mL; Dako, Denmark) for astrocytes and mouse anti-oligodendrocytes monoclonal antibody (1∶20000; Chemicon, Millipore, Billerica, USA) for oligodendrocytes. Incubation in the primary antibody was applied for 1 h at RT. After washing three times with PBS, cells were incubated in the rhodamine conjugated secondary antibody, including donkey anti mouse IgG (1∶200; Dako, Denmark), donkey anti goat (1∶200; Dako, Denmark), or donkey anti rabbit IgG (1∶200; Dako, Denmark). Slides were counterstained with DAPI (100 ng/mL; Dako, Denmark). All slides were examined under a fluorescence microscope (Olympus model BX51, Japan). The evaluation and quantification was done on 150 cells for each type of differentiation and repeated three times.

### *In vivo* Cellular MRI Demonstration

1500 SPION-labeled neurospheres were thoroughly washed, centrifuged, and resuspended in PBS, and then injected into the left ventricle (0.8 mm caudal, 1.5 mm lateral, and 2.9 mm ventral to bregma) of a rat brain with glioma. The glioma was caused by *in utero* exposure to ethyl-nitrourea (ENU). Three animals were used. The injection volume was 5 µl and the infusion rate was1 µl/min using a micro-infusion pump (Model 310; KD Scientific Inc., USA). Each animal was allowed to recover after the surgery before MRI scanning. The animal was imaged at baseline, day 1, day 7, and day 14. The rat was scanned on a 7-T spectrometer (PharmaScan 70/16, Bruker, Germany) with an active shielding gradient at 300 mT/m in 80 µs, capable of maximum 30 G/cm with a slew rate 3750 T/m/s. A volume coil with a 38 mm inner diameter was used for both signal excitation and receiving. The rat was fixed in a prone position during scanning. To ensure the same areas were selected for repeated imaging, each time the rat brain was aligned with several anatomical landmarks such as the anterior commissure and the midline. T2* weighted images (WI) and T2WI were repeatedly acquired before and after NPC transplantation. For T2*WI, a FLASH sequence was used with field of view (FOV) = 2.56×2.56 cm^2^, matrix size = 256×128, slice thickness = 1 mm, TR = 1200 ms, TE = 20 ms, flip angel = 30°, NEX = 12. The T2*WI was zero-filled to 256×256 to achieve an in-plane resolution of 100 µm×100 µm. For T2WI, a fast spin-echo sequence was used with FOV = 2.56×2.56 cm^2^, matrix size = 256×256, slice thickness = 1 mm, TR = 4500 ms, effective TE = 70 ms, echo train length = 8, and NEX = 8. The injection and tumor sites were chosen as regions of interest (ROIs). The signal-to-noise ratio of these areas were analyzed relative to that of the cortex serving as a control region as mean ± standard error. At the last time point of MRI, the rat was euthanized and then transcardially perfused with 4% phosphate buffered paraformaldehyde. Brains were removed from the cranium, kept in the same fixative overnight at 4°C, and sectioned at 20 µm. The transplanted cells were identified by PB staining counterstained by nuclear fast red.

## Results

### Labeling Efficiency Determined by the Incubation Time and Iron Concentrations

The NPCs were incubated with SPION in 0, 25, 50, 75, or 100 µg iron/ml for 0.5, 12, or 24 hrs. In Fig. 1, PB staining results showed that prolonged incubation and high iron concentrations resulted in more labeling. The optimized condition for labeling NPCs was 75 µg/ml over 48 hours, in which all cells were observed with PB staining in the cytoplasm. Lower concentrations were less efficient in labeling the cells while concentrations higher than 75 µg/ml (i.e. 100 µg/ml) tended to cause undesired SPION aggregation.

### Neurosphere Formation of SPION Labeled NPCs

**Figure 2:**
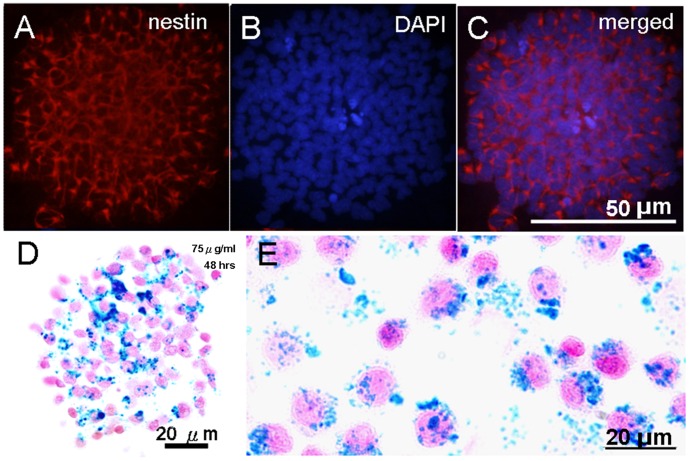
SPION-labeled NPCs formed neurospheres. (A) The neural progenitor identify confirmed by nestin immunostaining. (B) Nuclear staining by DAPI. (C) Merged nestin and DAPI staining. The majority of the cells within the sphere were nestin positive. (D) SPION labeling was examined by PB staining with nuclear fast red counterstain. (E) An enlarged view of dissociated SPION labeled NPCs stained with PB.

The SPION labeled NPCs formed neurospheres. The neurospheres formed by labeled NPCs coincided with those by unlabeled NPCs in the size (∼150 uM), shape (spherical), and time (∼7 days). Nestin immunostaining (Fig. 2A), DAPI (Fig. 2B), and the merged image (Fig. 2C) indicate the neural progenitor identity of the cells. The neurospheres were stained positive for PB (Fig. 2D). An enlarged view of the dissociated SPION labeled NPCs is shown in Fig. 2E.

### Intracellular Labeling Efficiency

**Figure 3:**
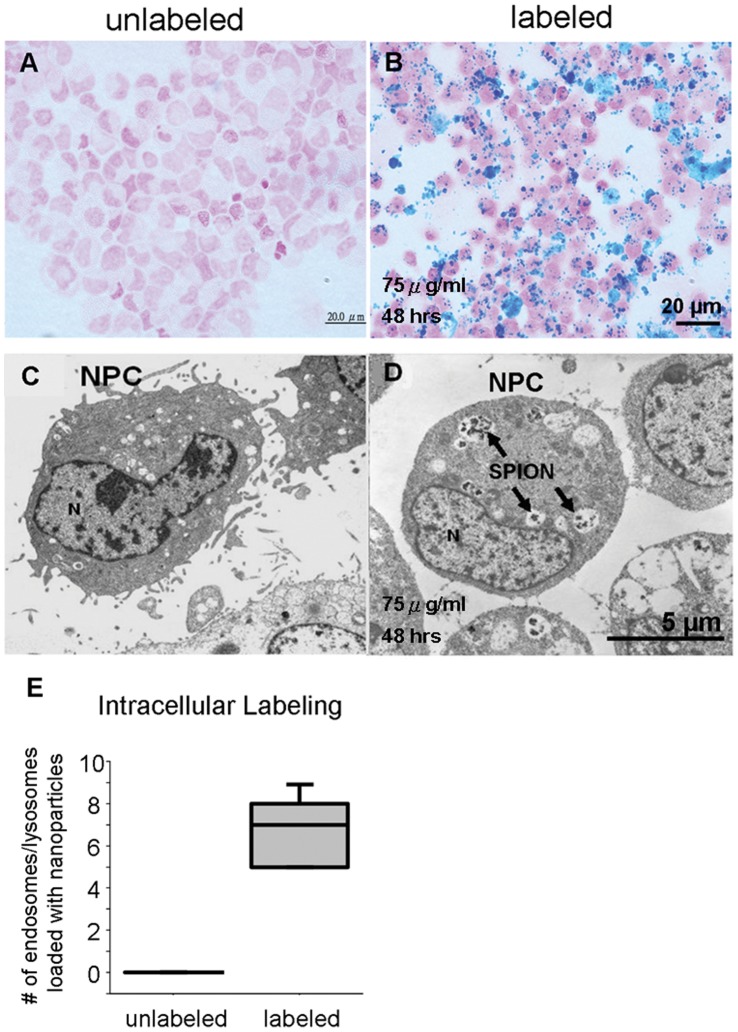
Intracellular labeling efficiency of simple SPION incubation. (A) Unlabeled NPCs stained with PB staining. (B) SPION-labeled NPCs. (C) Unlabeled NPCs under electronic microscopy. (D) SPION-labeled NPCs. Several endosomes or lysosomes loaded with SPION-like particles were observed in the cytoplasm, indicated by arrows. N indicates the nucleus. (E) Quantification of the loaded endosomes/lysosomes indicates the intracellular labeling efficiency of the simple incubation method.

PB staining was absent in unlabeled NPCs (Fig. 3A) but abundant in SPION labeled NPCs (Fig. 3B). When examining the PB staining at 100X, the counterstain dye (nuclear fast red) was found not only in the nuclei as pink but also in the cytoplasm as light pink. The cytoplasmic light pink color helped to define the cell-cell boundaries, and thus confirmed the intracellular localization of SPIONs. Electron microscopy reveals no appearance of nanoparticles in the cytoplasm of the unlabeled NPCs (Fig. 3C). By contrast, in the labeled NPCs, several vesicles loaded with particles likely to be SPIONs were observed (Fig. 3D). The vesicles resembled endosomes or lysosomes. Quantification of the loaded vesicles in the unlabeled and labeled NPCs is shown in Fig. 3E. Unlabeled cells have no SPION-loaded endo/lysosomes. Only unloaded endo/lysosomes were observed.

### Quantification of Iron Content

**Figure 4:**
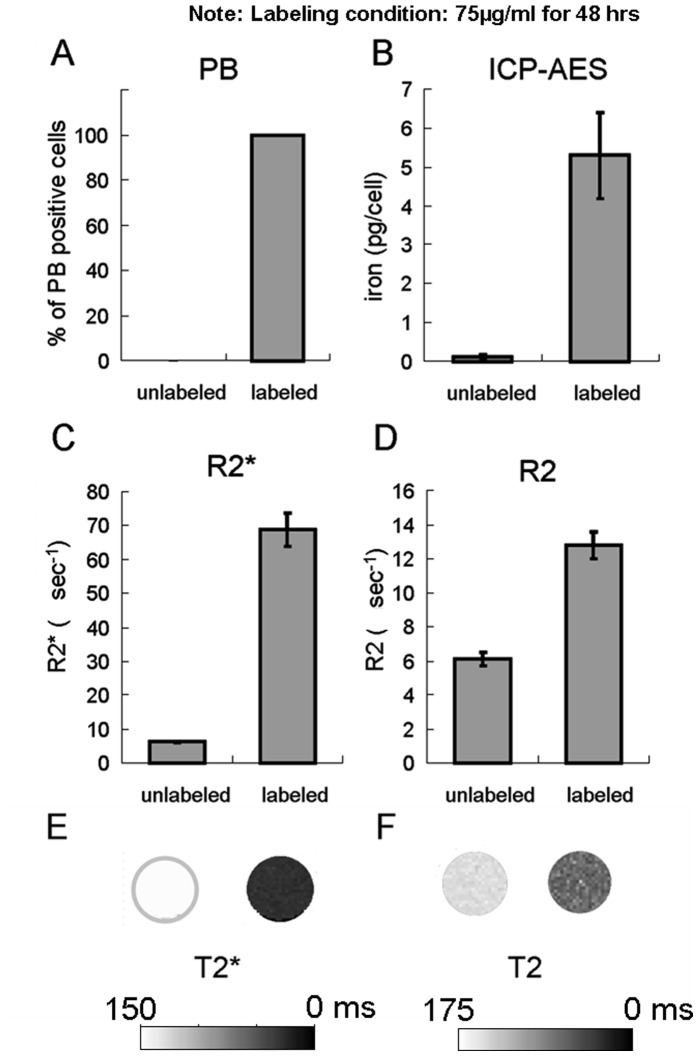
Examination of the iron content after simple SPION incubation with light microscopy, chemical analysis, and *in vitro* MRI. (A) No PB staining found in unlabeled cells while all the labeled ones were PB positive. (B) Little iron was detected in unlabeled cells while the labeled cells showed an averaged iron content of 5.3±1.1 pg/cell. (C) The R2* value was minimal in unlabeled cells while the labeled cells showed an averaged R2* value of 68.7±4.9 ms^−1^. (D) The R2 value was 6.1±0.4 ms^−1^ in unlabeled cells while the labeled cells showed an averaged R2 value of 12.8±0.8 ms^−1^. (E) The T2* map. (F) The T2 map.

The simple SPION incubation method resulted in 100% PB positive NPCs while no PB positive cells were seen in unlabeled NPCs (Fig. 4A). Chemical analysis of the iron content by ICP-AES indicated the averaged iron content was 5.3±1.1 pg/cell in the labeled NPCs and 0.1±0.06 pg/cell in the unlabeled ones (Fig. 4B). *In vitro* MRI relaxometry indicated that the R2* values were 6.2±0.2 ms^−1^ and 68.7±4.9 ms^−1^ for unlabeled and labeled NPCs, respectively (Fig. 4C). The R2 values were 6.1±0.4 ms^−1^ and 12.8±0.8 ms^−1^ for unlabeled and labeled NPCs, respectively (Fig. 4D). The T2* and T2 maps of the NPCs solidified in agarose are shown in Fig. 4E, and Fig. 4F. The labeled NPCs appeared as hypointensities on both of the images.

### No Changes in Cellular Viability, Oxidative Stress, and Apoptosis after SPION Labeling

**Figure 5:**
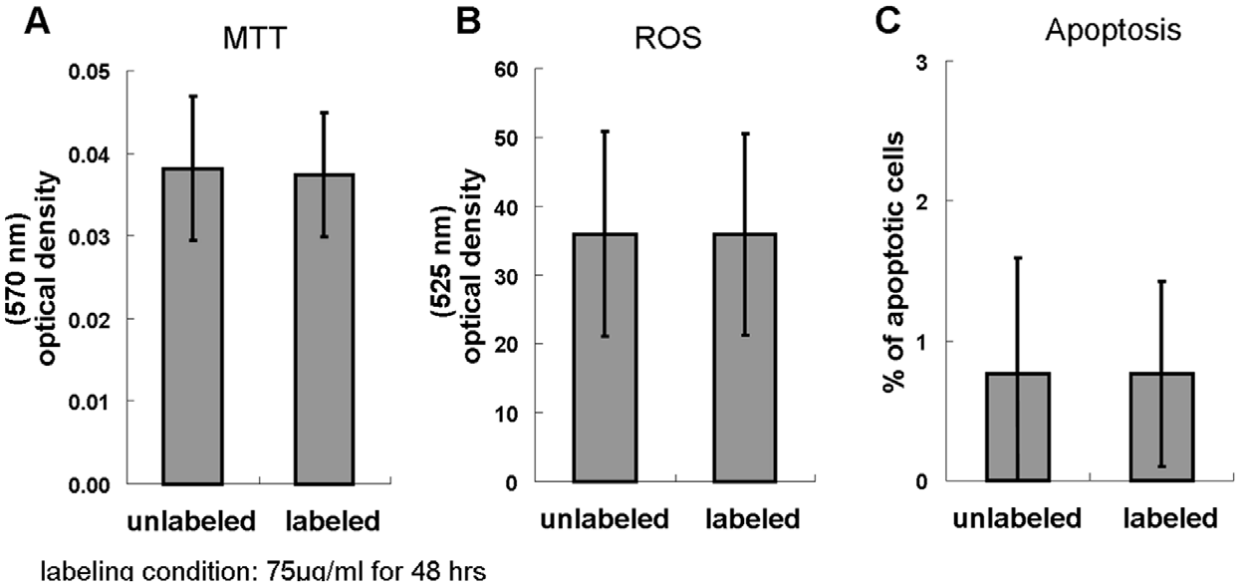
No adverse effects of the proposed method in fundamental cellular functions. (A) Comparable viability between unlabeled and labeled cells. (B) Comparable ROS production. (C) Comparable apoptosis.

Fig. 5 shows that there was no difference in formazan formation in the MTT viability assay, the ROS production, and the annexin-V positive percentage in the apoptosis test between the unlabeled and labeled NPCs.

### Neural Differentiation

**Figure 6:**
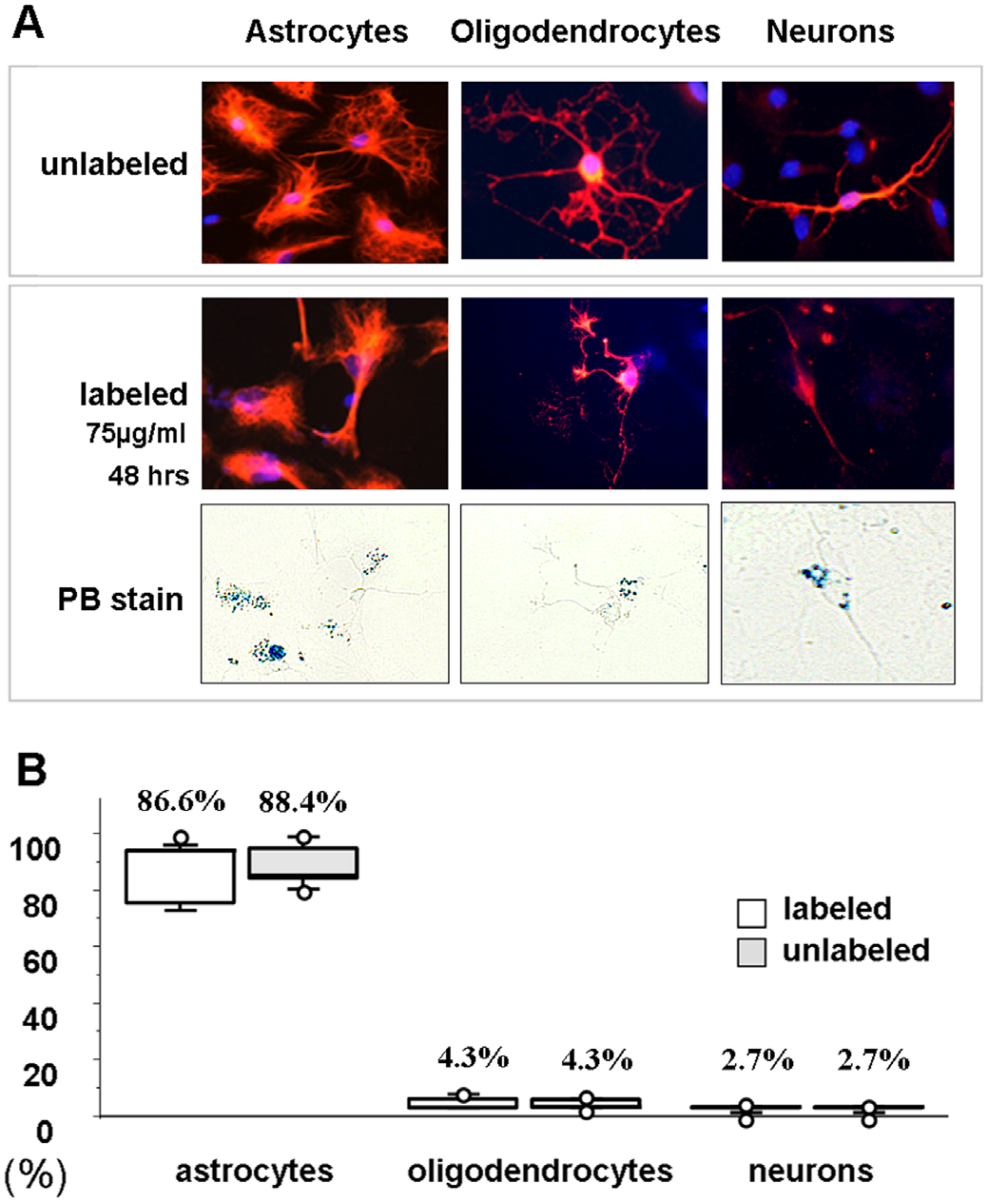
Comparison of the differentiation capacity between unlabeled and labeled NPCs. Following 48 hours of SPION incubation and 72 hours of induction to grow as astrocytes (anti-GFAP), oligodendrocytes (anti-oligodendrocytes) or neurons (anti-βIII- tubulin), both unlabeled and labeled NPCs exhibited similar differentiation patterns. (A) The immunofluorescent staining of astrocytes, oligodendrocyte, and neurons along with PB staining. (B) Quantitative analysis indicated labeling produced no differences in the cell type of neural differentiation.

The differentiation capacity of labeled NPCs was compared with unlabeled ones. Following 48 hours of SPION incubation and 72 hours of induction to grow as astrocytes, oligodendrocytes or neurons, both unlabeled and labeled NPCs exhibited similar differentiation patterns. Fig. 6A shows the immunofluorescent staining of astrocytes, oligodendrocyte, and neurons along with PB staining. In Fig. 6B, the quantitative analysis indicated no difference in the cell type of differentiation between unlabeled and labeled NPCs.

### Comparing Labeling at Different Iron Concentrations

Table 1 summarizes the labeling efficiency (PB positive staining), oxidative stress (ROS), and cell viability (MTT) of the labeling conditions of 25 µg/mL, 75 µg/mL, and 100 µg/mL for 48 hours. The numbers were expressed as the percentages of the labeled to the unlabeled cells examined simultaneously. The variations among the conditions were not statistically different. In addition, the induced differentiation of the three conditions was also comparable with that observed in the unlabeled cells.

**Table 1:** Labeling NPCs with only SPION at different concentrations.

	25 µg/mL	75 µg/mL	100 µg/mL
PB%	98%	100%	100%
ROS	104%	97%	107%
MTT	100%	102%	100%

* Numbers are expressed as the percentages of the labeled to the unlabeled cells examined simultaneously.

### *In vivo* MRI of SPION-labeled NPCs

**Figure 7:**
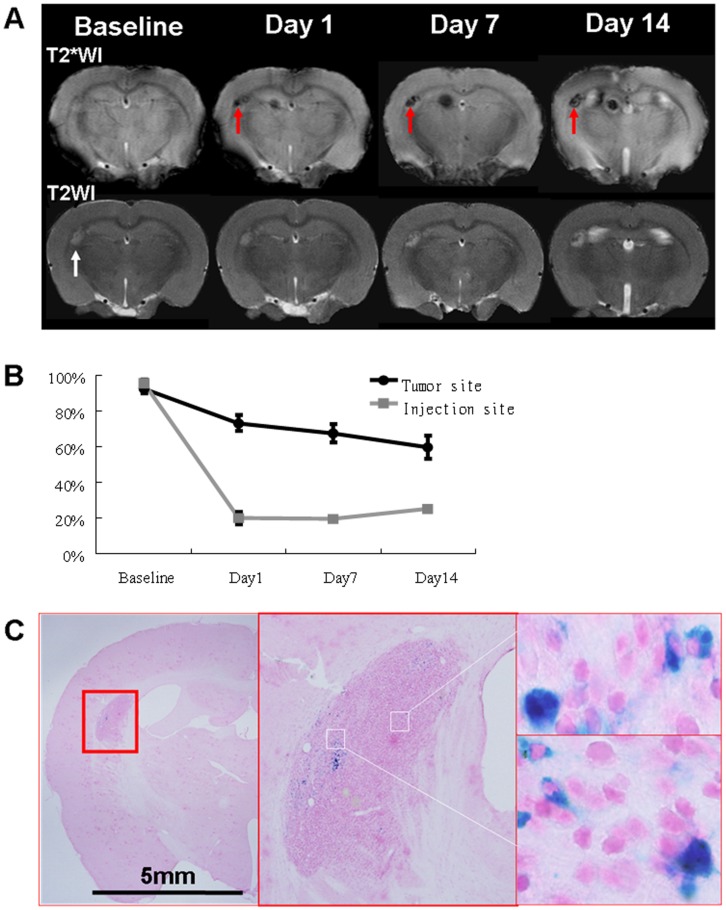
A demonstration *in vivo* cellular MRI using the simple labeling method. A rat with glioma was transplanted with SPION-labeled NPCs into the lateral ventricle, and T2WIs were used to track the migration and distribution of the transplanted NPCs. (A) T2WI acquired at baseline, on day 1, 7, and 14 after transplantation. Hypointensities were readily visible one day later, and persisted throughout the tracking duration. (B) Signals at the tumor or injection site in relation to a control area (the cortex) were plotted against time. (C) PB staining confirmed the relocation of SPION-labeled cells following transplantation. Histology was performed on brain tissues obtained on day 14 after transplantation. The white arrow indicates the location of the tumor whereas the red arrows indicate the hypointensity arising from the labeled NPCs. For tumors without injected NPCs, the signal patterns were different. Untreated ENU tumors exhibited only mild hypointensities likely associated with hemorrhage on T2WI and T2*WI.

The T2*WI and T2WI at baseline and at 1, 7, and 14 days after transplantation of SPION-labeled NPCs into the lateral ventricle of the rat with glioma are shown in Fig. 7A. The tumor was identified as a hyperintensity at baseline. One day following transplantation, the signal at the tumor site became hypointense and persisted throughout the tracking duration. Quantification of the signal at the tumor or injection site over time is shown in Fig. 7B. The tumor exhibited increasing hypointensity and the injection site remained the same hypointense. PB staining performed on the brain tissues obtained 14 days after transplantation indicated that iron was present in the tumor site, which should be responsible for the signal changes on T2*WI and T2WI (Fig. 7C). For tumors without injected NPCs, the signal patterns were different. Untreated ENU tumors exhibited only mild hypointensities associated with hemorrhage on T2WI and T2*WI (data not shown).

## Discussion

The current study found that simple SPION incubation without the addition of TAs is an efficient intracellular magnetic labeling method. Following 48 hours of incubation at 75 µg/ml, nearly all NPCs showed visible SPION intake. Evidence from light microscopy, electron microscopy, chemical analysis, and magnetic resonance imaging confirmed the effectiveness of the labeling. Additionally, biological assays showed that the labeled NPCs exhibited unaffected viability, oxidative stress, apoptosis, and differentiation, indicating the biosafety of this labeling methodology. In the demonstrated *in vivo* cellular MRI experiment, the hypointensities representing the SPION labeled NPCs remained observable throughout the entire tracking period.

TAs improve the loading of SPION into cells by modifying the surface charge of the particles. SPION have a negative surface charge that prevents them from efficiently adhering to cell membranes and hence from being internalized into cells [24]. Once the nanoparticles are complexed to cationic TAs, the altered surface charge allows nanoparticles to enter cells more readily [9]. However, our findings indicate that the electric charge present at the cellular interface is not the only factor affecting the loading efficiency of SPION. When the incubation is prolonged and the iron concentration is increased, the intracellular internalization of SPION is significantly improved. It has been shown that SPION labeling without the use of TAs led to upregulation of the transferrin receptors in the labeled cells [19]. This is thus speculated to be the mechanism via which SPION is loaded intracellularly by the simple incubation method. Indeed, transferrin receptors are known to play a role in either the SPION-TA complex [25] or the simple labeling method [19]. The iron level rendered by either method is a key determinant of the dynamic uptake of iron via the transferrin receptors.

Table 2 summarizes the findings from the literature in SPION labeling without and with the transfection agents. Although plain labeling was reported before, most studies found it unsatisfactory. It was either associated with adverse effects on viability and growth [3] or suboptimal labeling efficiency [10], [20], [26]. To the sharp contrast, the SPION-TA method received widespread support from several groups, and thus became the leading labeling method for cellular MRI. However, our study revisited the plain labeling approach by optimizing the incubation conditions and found the method unexpectedly efficient. An additional experiment was carried out to follow the effects of the simple incubation method or the SPION-TA one for one week. As indicated in Table 3, the two approaches were comparable in terms of the labeling efficiency and cell viability over the seven day time course. But significant transient higher ROS were observed following the SPION-TA complex method, which was likely associated with a surge in the degradation of the complex.

**Table 2:** SPION labeling with and without TAs in the literature.

SPION only	cell	concentration	time	results
Sipe, 1999	lymphocytes/monocyte	4.17 mg/mL	4 hr	hypointensity observable on T2WI
Dunning, 2004	Schwann cells/ensheathing cells	2 mg/mL	48 hr	hypointensity observable on T2WI
		0.5 mg/mL	48 hr	undetectable
Schafer, 2007	mesenchymal stem cells	200 µg/mL	15 hr	24.7 pg/cell
Neri, 2008	human neural progenitor cells	800 µg Fe/ml	72 hr	100%
Jing, 2008	mesenchymal stem cells	50 µg/ml	2 hr	∼38% labeling
Kunzmann, 2011	immune cells	50 µg/ml	2 hr	sparse on EM
		50 µug/ml	24 hr	observable on EM
SPION+TA				
Frank, 2003	mesenchymal stem cells	25 µg Fe/mL+TA	2 hr	7.6–30.1 pg/cell
Arbab, 2003	T-cells	50 µg Fe/mL+TA	3 hr	100% PB positive; 1.53 0.26 pg/cell
	mesenchymal stem cells	25 µg Fe/mL+TA	3 hr	100% PB positive; 12.61 2.47 pg/cell
Arbab, 2004	mesenchymal stem cells	50 µg Fe/mL+TA	2–3 hr	100% PB positive; ∼10 pg/cell; R2>100 sec-1
Miyoshi, 2005	neural stem cells	250 µg Fe/mL+TA	6 hr	5–6 pg/cell
Schafer, 2007	mesenchymal stem cells	60 µg Fe/mL+TA	4 hr	76 pg/cell
Suzuki, 2007	embryonic stem cells	50 µg Fe/ml +TA	12–24 hr	8.9–11.9 pg/cell; dephaseing signal 22.5–37.5 mm2
Neri, 2007	human neural progenitor cell line	400 µg Fe/ml+TA	48 hr	100%
		25 µg Fe/ml +TA	24 hr	>80%
Jing, 2008	mesenchymal stem cells	50 µg Fe/ml +TA	2 hr	>90% labeling

**Table 3:** Comparison between labeling with SPION only or SPION+PLL during the following week.

		D1	D5	D7
PB%	SPION only (75 ug/mL)	99.5%	93.5%	78.5%
	SPION+PLL (25 ug/mL+PLL)	99.4%	94.5%	79.0%
ROS	SPION only	128.2%	123.5%	116.7%
	SPION+PLL	127.7%	146.7%*	118.4%
MTT	SPION only	98.5%	101.5%	98.8%
	SPION+PLL	91.9%	100.0%	98.8%

† Numbers are expressed as the percentages of the labeled to the unlabeled cells examined simultaneously.

The SPION-TA complex method often employs low iron concentrations (25 µg iron/ml) and short incubation time (2 hours). The SPION dosage was rather low as compared to previous approaches that used SPION concentrations without TA on the order of thousands [11], [26] or hundreds of µg per ml [19]. Our study found that high labeling effectiveness can also be achieved without the TA or very high SPION concentration. Prolonged incubation with moderate SPION dosage (i.e. 75 µg iron/ml for 48 hours) would be a straightforward regimen to avoid the potential adverse effects associated with TA or excessive intracellular iron levels.

Biosafety is crucial to the applicability of a magnetic cell labeling method in clinical settings. As mentioned earlier, SPION complexed with TAs undergo degradation after entry into intracellular spaces [14]. Dissociation of the complexes leads to the labeled cells being exposed to the toxic effects of both TAs and unbound iron [17]. The mechanisms underlying the toxicity of TAs are not well understood, but apoptosis via the activation of protein kinases may be involved [16]. Iron in a dissociated or free state is considered to be detrimental because it stimulates the conversion of hydrogen peroxide into free radicals that attack cellular organelles, including DNA [27]. A reported transient increase in ROS in cells labeled with SPION for a long time using a TA corresponds with this view [5]. In contrast, the simple labeling method used in the current study is not affected by the dissociation problems, which coincides with our observation that the fundamental biological properties of the labeled cells are largely preserved without adverse effects.

SPION complexed to protamine sulfate (PS) is a labeling method that involves the use of two clinically approved agents [6]. In this method, PS (used as the TA) is a natural peptide that is approved by the FDA to be used as an antidote to heparin anticoagulation, and the method offers good labeling efficiency and preserves the biological properties of the labeled cells [25]. In addition, because it uses only clinically approved substances, it is more applicable to human use than other proposed labeling methods. Nevertheless, as opposed to simple incubation, the PS-SPION method is admittedly less straightforward. When employing the PS-SPION method, the ratio of PS-to-SPION needs to be carefully optimized to obtain reliable relaxation rates. It was shown that R2 values were unusually low when the complexes had low zeta potentials (i.e.<|±10 mV|), suggesting instability or coagulation of the PS-SPION complexes (low zeta potentials are related to low stability of colloids.). This may be associated with unexpected actions by PS at the paramagnetic sites on the iron core surface, causing fluctuations in the relaxation rates [6].

The present study used a simple labeling method to load SPION into NPCs. This method avoids the use of TAs, and therefore is not affected by the potential adverse effects of TAs. When TAs are used, labeling conditions need to be well controlled to prevent nanoparticles from precipitating or forming aggregates with TAs. The labeling procedures employed in the present study are relatively straightforward, so the method can be readily applied and repeated with high reliability. Most importantly, simple SPION labeling produced no adverse effects on the biological properties of labeled cells. Since SPION is already a clinical approved contrast agent that can be used in humans, the current labeling method has the considerable advantage of already being available for clinical use. An *in vivo* application was also evaluated in this study, in which labeled cells were implanted into glioma-bearing rats and longitudinally tracked by MRI. Labeled cells were found at the site of injury 1 day after implantation, supporting the effectiveness of the current labeling method for tracking the distribution and migration of NPCs using MRI.
